# Expression of Human L-Dopa Decarboxylase (DDC) under Conditions of Oxidative Stress

**DOI:** 10.3390/cimb45120635

**Published:** 2023-12-16

**Authors:** Nikolaos S. Lotsios, Nikolaos Arvanitis, Alexandros G. Charonitakis, George Mpekoulis, Efseveia Frakolaki, Niki Vassilaki, Diamantis C. Sideris, Dido Vassilacopoulou

**Affiliations:** 1 Section of Biochemistry and Molecular Biology, Department of Biology, National and Kapodistrian University of Athens, 15701 Athens, Greece; n.lotsios96@gmail.com (N.S.L.); narvan@biol.uoa.gr (N.A.); alexharonit@yahoo.gr (A.G.C.); dsideris@biol.uoa.gr (D.C.S.); 2 Laboratory of Molecular Virology, Hellenic Pasteur Institute (HPI), 11521 Athens, Greece; g.mpekoulis@pasteur.gr (G.M.); nikiv@pasteur.gr (N.V.)

**Keywords:** L-Dopa decarboxylase, oxidative stress, H_2_O_2_, apoptosis, neural and non-neural cells

## Abstract

Oxidative stress is known to influence mRNA levels, translation, and proteolysis. The importance of oxidative stress has been demonstrated in several human diseases, including neurodegenerative disorders. L-Dopa decarboxylase (DDC) is the enzyme that converts L-Dopa to dopamine (DA). In spite of a large number of studies, little is known about the biological significance of the enzyme under physiological and pathological conditions. Here, we investigated the relationship between DDC expression and oxidative stress in human neural and non-neural cells. Oxidative stress was induced by treatment with H_2_O_2_. Our data indicated that mRNA and protein expression of DDC was enhanced or remained stable under conditions of ROS induction, despite degradation of total RNA and increased cytotoxicity and apoptosis. Moreover, DDC silencing caused an increase in the H_2_O_2_-induced cytotoxicity. The current study suggests that DDC is involved in the mechanisms of oxidative stress.

## 1. Introduction

Redox homeostasis is the maintenance of a balance between oxidative and reductive molecules. Oxidative stress results either from an increase in the production of reactive oxygen species (ROS) or the breakdown of cellular antioxidant systems that disrupt the balance [1]. The mitochondrion is the primary source of ROS. Under normal conditions, ROS are produced as a byproduct of aerobic metabolism [2]. The oxidation of biomolecules (nucleic acids, proteins, and lipids) has been associated with overproduction of ROS [3,4,5]. Oxidative stress has a major impact on the development of several neurodegenerative diseases, including Parkinson’s (PD) and Alzheimer’s (AD), various cancers, and cardiovascular disorders [6].

L-Dopa decarboxylase (DDC; EC 4.1.1.28) is a pyridoxine-5-phosphate-requiring enzyme, which decarboxylates L-3,4-dihydroxyphenylalanine (L-Dopa) and 5-hydroxytryptophan (5-HTP) converting them to dopamine (DA) and serotonin (5-HT), respectively [7]. DDC is additionally known as aromatic L-amino acid decarboxylase (AADC), since it is thought to decarboxylate other aromatic L-amino acids in mammalian tissues [8,9,10]. The human *DDC* gene is present in a single copy on chromosome 7 (7p12.1–7p12.3) [11]. Distinct mRNA transcripts, neuronal and nonneuronal, encoding the same proteins, are formed due to alternative splicing events within the 5’ untranslated regions (5’ UTR) [12,13,14,15]. The neuronal mRNA transcript has also been observed in non-neuronal tissues in humans such as placenta, kidney, leukocytes, and liver cells [14,16,17,18,19]. An alternative transcript lacking exon 3, referred to as DDC-3, has been identified and its expression is the result of an alternative splicing event in the coding region [20]. In addition, a novel human DDC mRNA variant containing an alternative exon 10 and missing exon 10 to exon 15 of the full-length transcript, thus encoding a shorter 338 amino acid polypeptide, has been identified by our research team [21]. It is emphasized that the biological role of the DDC protein isoforms is still completely unknown. Several other variants of human DDC mRNA produced due to alternative splicing have been detected using next generation sequencing. Many of these presumably encode novel protein isoforms [22]. The significance of each DDC expression pattern remains unclear. The above results highlight the complexity of the regulation of human DDC expression.

DDC expression in the nervous system is important for neurotransmission [23]. Its involvement in neurodegeneration, signal transduction, viral infections, and apoptosis has been recently investigated. In particular, DDC was shown to suppress tau-induced toxicity in a study where deletion of the *Caenorhabditis elegans* DDC homolog gene, *bas-1*, resulted in decreased tau phosphorylation and suppressed tau toxicity [24]. A study on Parkinson’s disease patients demonstrated that *DDC* gene polymorphisms modulate the motor response to L-Dopa [25], possibly by altering available dopamine in the central nervous system [25]. Moreover, the motor response to L-Dopa was shown to be modulated by the single nucleotide polymorphism DDC rs921451 [26]. A recent study by our team, in human hepatocytes, kidney, and neural cells, documented the association of the enzyme with phosphatidylinositol 4,5-biphosphate 3-kinase (PI3K) [19]. Our group has also revealed that DDC is a critical factor in the life cycles of *Flaviviridae* viruses DENV and HCV [27]. Recent research has also illustrated how DDC is involved in apoptotic processes [28].

Because of the emerging complex role of DDC in physiological and pathological processes and because of the observation that human DDC is implicated in apoptosis, we sought to examine the expression patterns of the enzyme under conditions of severe oxidative stress in human cells of neural and non-neural origin and the effect of the DDC-silencing on H_2_O_2_-induced cytotoxicity.

## 2. Materials and Methods

### 2.1. Cell Culture

SH-SY5Y (human neuroblastoma) and HEK-293 (human embryonic kidney) cell lines were obtained from the American Type Culture Collection (ATCC, Manassas, VA, USA). Prof. C. Rice from The Rockefeller University (New York, NY, USA) kindly contributed the Huh7.5 cell line [29]. The medium used for cell culture was Dublecco’s Modified Eagle’s Medium (DMEM) with high glucose (25 mM), supplemented with 10% (*v*/*v*) Fetal Bovine Serum (FBS, Biosera, Cholet, France) heat-inactivated, L-glutamine (4 mM) (Biosera), 100 μg/mL streptomycin (Biosera) and 100 U/mL penicillin (Biosera).

### 2.2. Establishment of a Huh7.5-Based Cell Line with Stable Silencing of DDC

The generation of the DDC-silenced stable cell line was conducted via lentiviral transduction in Huh7.5 cells. To produce lentiviral particles, HEK-293 cells were co-transfected, using the transfection reagent lipofectamine (Invitrogen, Carlsbad, CA, USA), with three plasmids, as described elsewhere [30]: (1) pczVSV-G, expressing the envelope glycoprotein of vesicular stomatitis virus [31]; (2) pRCMVΔR8.74 [32], expressing the HIV-1 Gag-Pol; (3) The negative control (shControl) or psi-LVRH1GP/shDDC (shDDC) vectors. Culture medium containing 2 μg/mL puromycin was utilized for the positive selection of the transduced cells. DDC mRNA and protein were quantified to confirm DDC silencing in the shDDC-expressing cells.

### 2.3. Assessment of Reactive Oxygen Species Levels

ROS levels were measured using the H_2_DCFDA (Invitrogen, Carlsbad, CA, USA) indicator via flow cytometry as previously described [33]. Cell cultures were treated using increasing concentrations of H_2_O_2_ (Sigma-Aldrich, Burlington, MA, USA) for 30 and 60 min. Subsequently, cells were incubated in the presence of 5 μM of H_2_DCFDA for 30 min. Untreated cells were used as controls. Following centrifugation, samples were transferred to the FACS Calibur Cytometer (Becton-Dickinson, Franklin Lakes, NJ, USA), and 10,000 cells were used in each experiment. The obtained data were evaluated via the use of Flowing Software Version 2 (University of Turku, Turku, Finland).

### 2.4. Detection of Cytotoxicity and Apoptosis Levels

The Trypan Blue exclusion assay was implemented to assess cytotoxicity levels. Percentages were calculated as previously described [34]. Alternatively, the ViaLight HS BioAssay kit (Cat. Number: LT 07-221, Lonza, Basel, Switzerland) was used to quantify the intracellular ATP (energy) content in a GloMax 20/20 single-tube luminometer (Promega Corporation, Madison, WI, USA), according to the manufacturer’s instructions. The results were normalized using the total protein amounts. The data obtained were presented as the average of a minimum of three separate trials. Apoptotic cell death was evaluated by performing the diphenylamine method. The apoptotic levels were calculated by comparing the percentage of DNA fragmentation to the total amount of DNA [35]. The results presented are the average of three different experiments.

### 2.5. RNA Quantification by Reverse Transcription-Quantitative PCR (RT-qPCR)

The TRIzol reagent (Ambion, Foster city, CA, USA) was used to extract total RNA in accordance with the manufacturer’s instructions. Total RNA was visualized using 1% agarose gel electrophoresis. The Moloney murine leukemia virus reverse transcriptase (NEB, Ipswich, MA, USA) and oligo (dT) primers were then used to create cDNAs. The Luna Universal qPCR Master Mix (NEB) was used for real-time quantitative PCR (qPCR). A primer pair specific to exons 10–12 of the full-length DDC mRNA (DDC-F: 5′-GAACAGACTTAACGGGAGCCTTT-3′ and DDC-R: 5′-AATGCCGGTAGTCAGTGATAAGC-3′) was utilized to measure DDC expression. The specific primers HO-1 F (5′-ATGACACCAAGGACCAGAGC-3′) and HO-1 R (5′-GTGTAAGGACCCATCGGAGA-3′) were utilized to quantify the amounts of HO-1 mRNA. To normalize the results, the housekeeping gene 14-3-3 zeta polypeptide (YWHAZ) mRNA quantity was employed, using the primer pair YWHAZ-F: 5′-GCTGGTGATGACAAGAAAGG-3′ and YWHAZ-R: 5′-GGATGTGTTGGTTGCATTTCCT-3′. The samples were run in triplicate and then analyzed using the comparative CT method 2^−ΔΔCT^ [36].

### 2.6. Determination of Protein Concentration

Total protein concentration was measured following the Bradford method, using a standard Bovine Serum Albumin (BSA) [37].

### 2.7. Gel Electrophoresis and Western Blot Analysis

SDS-PAGE was carried out using a 12% polyacrylamide slab gel in the “Bio-Rad Mini Protean II” electrophoresis apparatus, as previously described [38]. The pre-stained protein markers used as MW standards were the BlueStar (Nippon Genetics, Duren, Germany). Electrophoresis was performed at 120 V for 100 min (room temperature). Samples were then blotted onto nitrocellulose [39]. DDC immunodetection was performed as described [40] using rabbit polyclonal antibody against human anti-C-terminal DDC prepared by our laboratory. DDC on the nitrocellulose membrane was detected using the chromogenic alkaline phosphate detection method (BCIP/NBT substrate). The intensity of the bands was measured using Actin (Millipore, Burlington, MA, USA) as a loading control. Quantitative analyses were performed via densitometry using ImageJ software version 1.53 (NIH, Bethesda, MD, USA).

### 2.8. Statistical Analysis

The mean value ± standard deviation (SD) of at least three independent experiments is represented by bars in all diagrams. Differences in ROS, cytotoxicity, apoptosis, ATP levels, DDC and HO-1 mRNA expression, and DDC protein levels were evaluated by performing a one-way ANOVA with Bonferroni’s post hoc test in the statistical software GraphPad Prism version 8.4.3 (San Diego, CA, USA). Every *p*-value is two-sided, and values *p* < 0.05 were deemed significant.

## 3. Results

### 3.1. Investigation of the Effect of H_2_O_2_ on the Production of ROS and the Expression of DDC Protein

**Figure 1:**
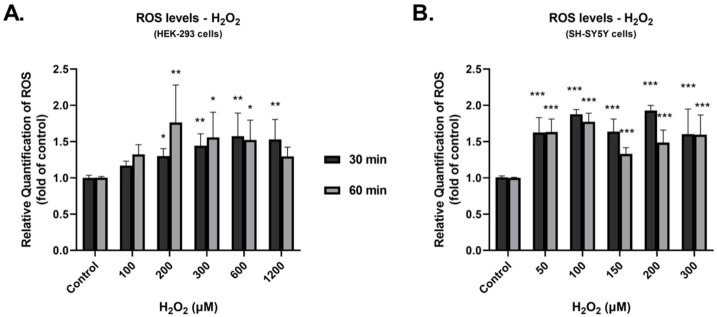
Effect of H_2_O_2_ on the production of reactive oxygen species (ROS) in HEK-293 (**A**) and SH-SY5Y (**B**) cells. Relative quantification of ROS levels in HEK-293 and SH-SY5Y cells following treatment with H_2_O_2_ for 30 (Black bar) and 60 min (Grey bar) at the indicated concentrations, as compared to untreated controls. Bars depict mean values of three separate experiments. Error bars indicate standard deviations. * *p* < 0.05, ** *p* < 0.01, and *** *p* < 0.001 vs. control.

We attempted to study the impact of H_2_O_2_ on the induction of oxidative stress and the expression of the full-length isoform of human DDC. Experiments were performed with the human cell lines neuroblastoma SH-SY5Y and embryonic kidney HEK-293. SH-SY5Y cells were less resistant to oxidative conditions and exhibited a higher percentage of cell death compared with HEK-293 cells. For this reason, different H_2_O_2_ concentrations were used for the two cell lines studied. First, we investigated the impact of H_2_O_2_ on the production of reactive oxygen species (ROS). SH-SY5Y and HEK-293 cells were incubated with 50–300 μM and 100–1200 μM H_2_O_2_ for 30 and 60 min, respectively. After exposure, treated and control cells were maintained for 30 min under 5 μM of the ROS indicator H_2_DCFDA. Production of ROS increased in both cell lines (Figure 1), as shown by flow cytometry.

For HEK-293 cells (Figure 1A), we detected a 1.6 peak-fold ROS increase at 600 μM H_2_O_2_ (*p* < 0.01) following 30 min exposure and a 1.8 peak-fold increase at 200 μM H_2_O_2_ (*p* < 0.01) following 60 min exposure. For SH-SY5Y cells (Figure 1B), a 1.9 peak-fold ROS increase was observed at 200 μM H_2_O_2_ (*p* < 0.001) following 30 min exposure and 1.8 at 100 μM H_2_O_2_ (*p* < 0.001) following 60 min exposure.

**Figure 2:**
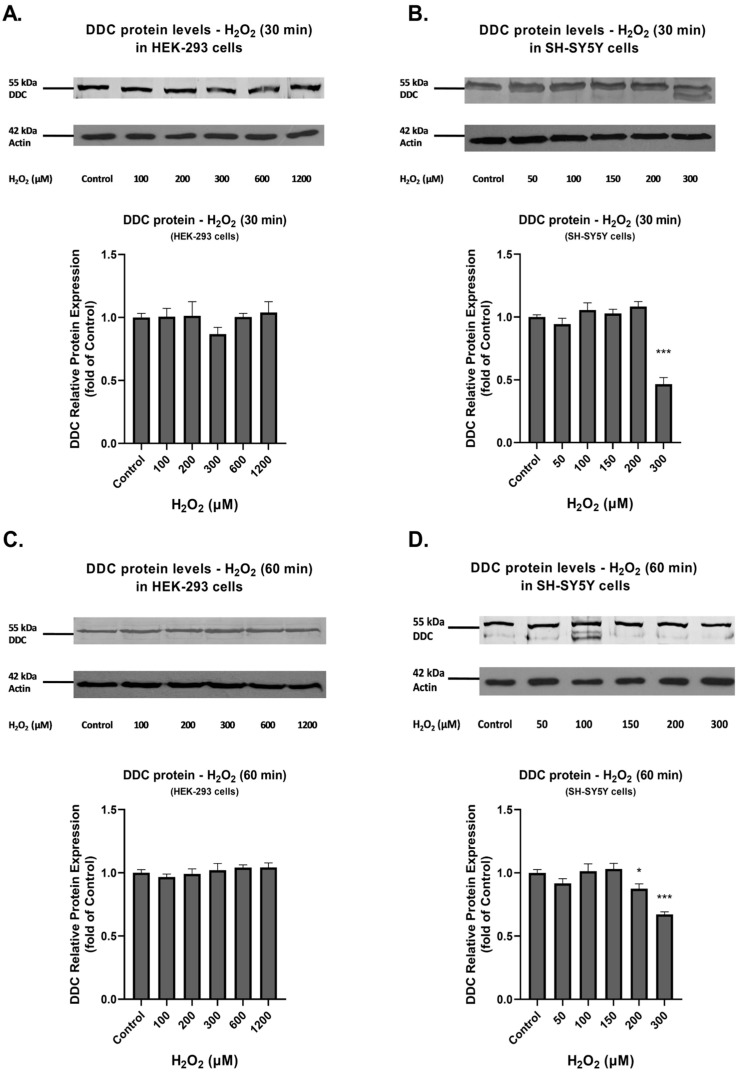
Effect of H_2_O_2_ on the expression of DDC protein in HEK-293 (**A**,**C**) and SH-SY5Y (**B**,**D**) cells. Western blot analysis performed with anti-DDC and anti-Actin antibodies in HEK-293 and SH-SY5Y cell lysates, following H_2_O_2_ exposure for 30 and 60 min at the indicated concentrations. Actin was used as the loading control. Graphs represent relative DDC protein expression against Actin. Bars depict mean values of two separate experiments. Error bars indicate standard deviations. * *p* < 0.05 and *** *p* < 0.001 vs. control.

DDC protein expression under identical conditions is shown in Figure 2. Our results indicate that, in general, the observed increases in production levels of ROS did not affect full-length DDC expression at the time points tested. In SH-SY5Y cells, DDC protein expression presented a significant decrease at 300 μM following 30 min exposure to H_2_O_2_ (Figure 2B, *p* < 0.001), as wells as at 200 μM and 300 μΜ H_2_O_2_ following 60 min exposure (Figure 2D; *p* < 0.05 and *p* < 0.001).

### 3.2. DDC mRNA and Protein Expression during Prolonged Exposure to Oxidative Conditions

On the basis of the above results indicating stability of DDC protein expression under increased ROS production, we examined DDC expression patterns under conditions of sustained and severe oxidative stress in HEK-293, SH-SY5Y, and the hepatoma Huh7.5 cells. We examined the mRNA and protein levels of DDC and the extent of cytotoxicity and apoptosis after 24 h of exposure. Different H_2_O_2_ concentrations were required for detecting cell viability alterations in the three cell lines because of their varying levels of resistance to oxidative stress.

**Figure 3:**
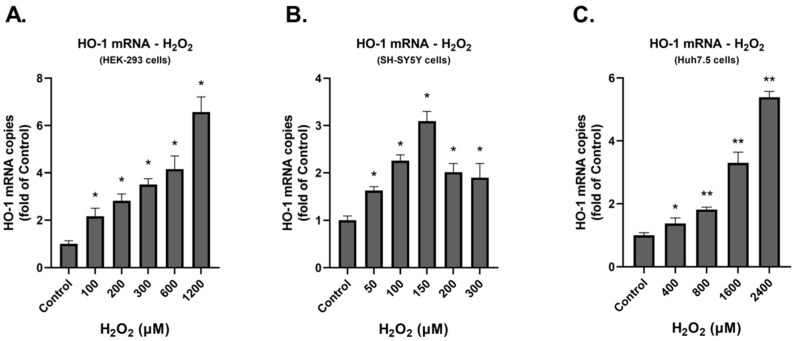
RT-qPCR quantification of HO-1 mRNA levels in HEK-293 (**A**), SH-SY5Y (**B**) and Huh7.5 (**C**) cells that were treated with H_2_O_2_ or were mock-treated (control) for 24 h. Values in control (mock-treated) cells were set to one. The mean values ± standard deviations from three independent experiments in triplicate are featured. * *p* < 0.05 and ** *p* < 0.001 vs. control.

First, to verify the induction of oxidative stress upon the prolonged H_2_O_2_ treatment, we evaluated the antioxidant gene heme oxygenase-1 (HO-1) expression in the three cell lines. Specifically, the cells were seeded in 12-well plates and 24 h later were treated with the appropriate concentrations of H_2_O_2_. Twenty-four hours post treatment total RNA was extracted from cells and the mRNA levels of HO-1 were determined. We observed that in HEK-293 and Huh7.5 cells HO-1 mRNA was increased in a dose-dependent manner (Figure 3). HO-1 expression was also elevated in SH-SY5Y cells upon treatment with H_2_O_2_, with the most substantial increase being observed at the concentration of 150 μΜ.

**Figure 4:**
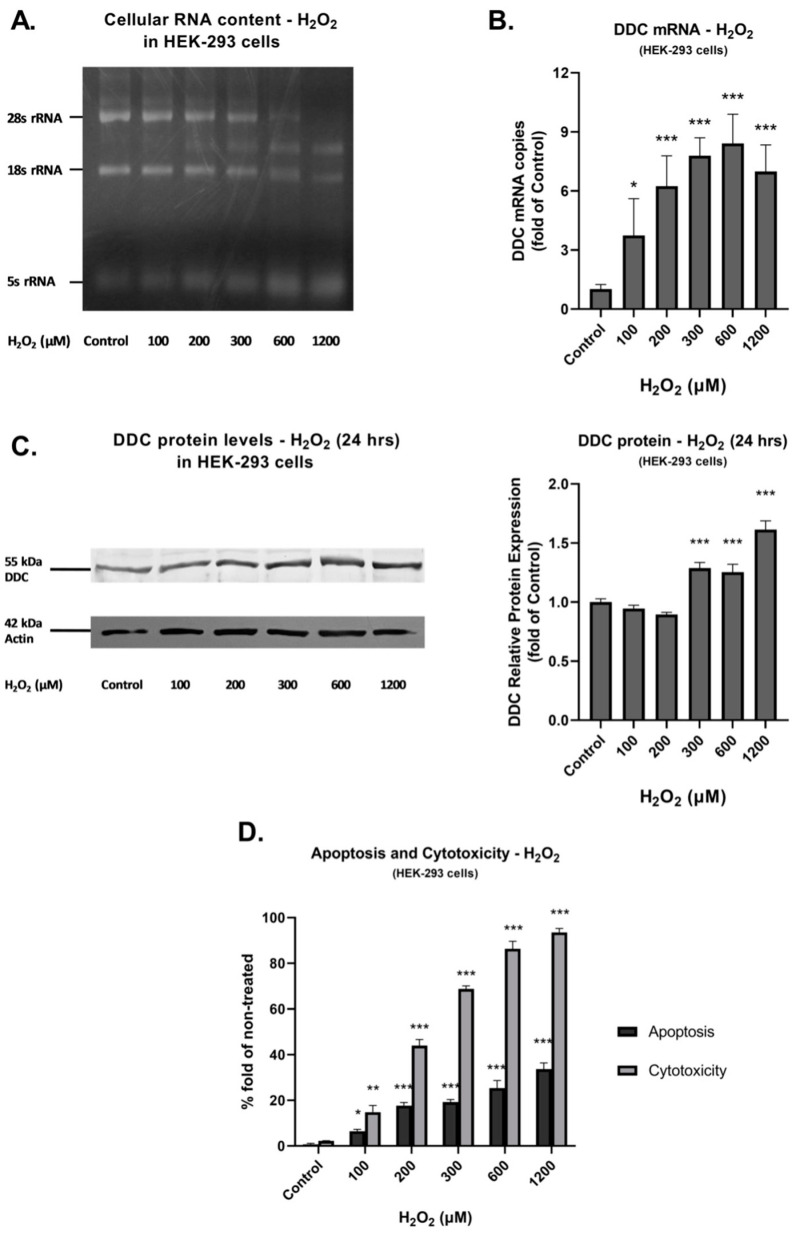
DDC expression, cytotoxicity, and apoptosis under conditions of prolonged oxidative stress in HEK-293 cells. (**A**) Agarose gel electrophoresis of extracted total RNA from cells exposed to H_2_O_2_ at the indicated concentrations for 24 h. (**B**) Relative DDC mRNA expression following exposure to H_2_O_2_ at the indicated concentrations for 24 h. Fold increase of DDC mRNA expression was measured using RT-qPCR in comparison to control (mock-treated) cells. Bars represent mean values from three separate experiments. Error bars represent standard deviation. * *p* < 0.05 and *** *p* < 0.001 vs. control. (**C**) Western blot analysis performed using anti-DDC and anti-Actin antibodies in HEK-293 cell lysates, following H_2_O_2_ exposure at the indicated concentrations for 24 h. Actin was used as the loading control. Graphs represent relative DDC protein expression against Actin. Bars depict mean values of three separate experiments. Error bars indicate standard deviations. *** *p* < 0.001 vs. control. (**D**) Effect of H_2_O_2_ on cytotoxicity (Grey bar) and apoptosis (Black bar): HEK-293 cells were exposed to H_2_O_2_ at the indicated concentrations for 24 h. Values presented are the percentages of cytotoxicity or apoptosis. Bars represent mean values from three separate experiments. Error bars represent standard deviation. * *p* < 0.05, ** *p* < 0.01, and *** *p* < 0.001 vs. control.

Subsequently, we examined whether the detected induction of oxidative conditions affects DDC expression. Figure 4 depicts obtained results for HEK-293 cells. As presented in Figure 4A, the increase in hydrogen peroxide concentration, although resulting in the degradation of 28S and 18S rRNAs starting at 300 μM H_2_O_2_, caused an elevation in DDC mRNA expression (Figure 4B). Specifically, it was found that DDC mRNA expression was upregulated in response to H_2_O_2_, with an increase ranging from 3.6-fold at 100 μM H_2_O_2_ to 8.2 at 600 μM H_2_O_2_. When DDC protein expression was examined, as depicted in Figure 4C, the obtained results demonstrated that full-length human DDC expression remained stable. Even more, in the case of exposure to 600 μM and 1200 μM H_2_O_2_, an increase in DDC protein levels was revealed. Finally, as depicted in Figure 4D, both cytotoxicity and apoptosis levels were induced in a dose-dependent manner. Peak cytotoxicity and apoptosis percentages were calculated at 1200 μM H_2_O_2_ corresponding to 93.5% (*p* < 0.001) and 33.6% (*p* < 0.001), respectively.

**Figure 5:**
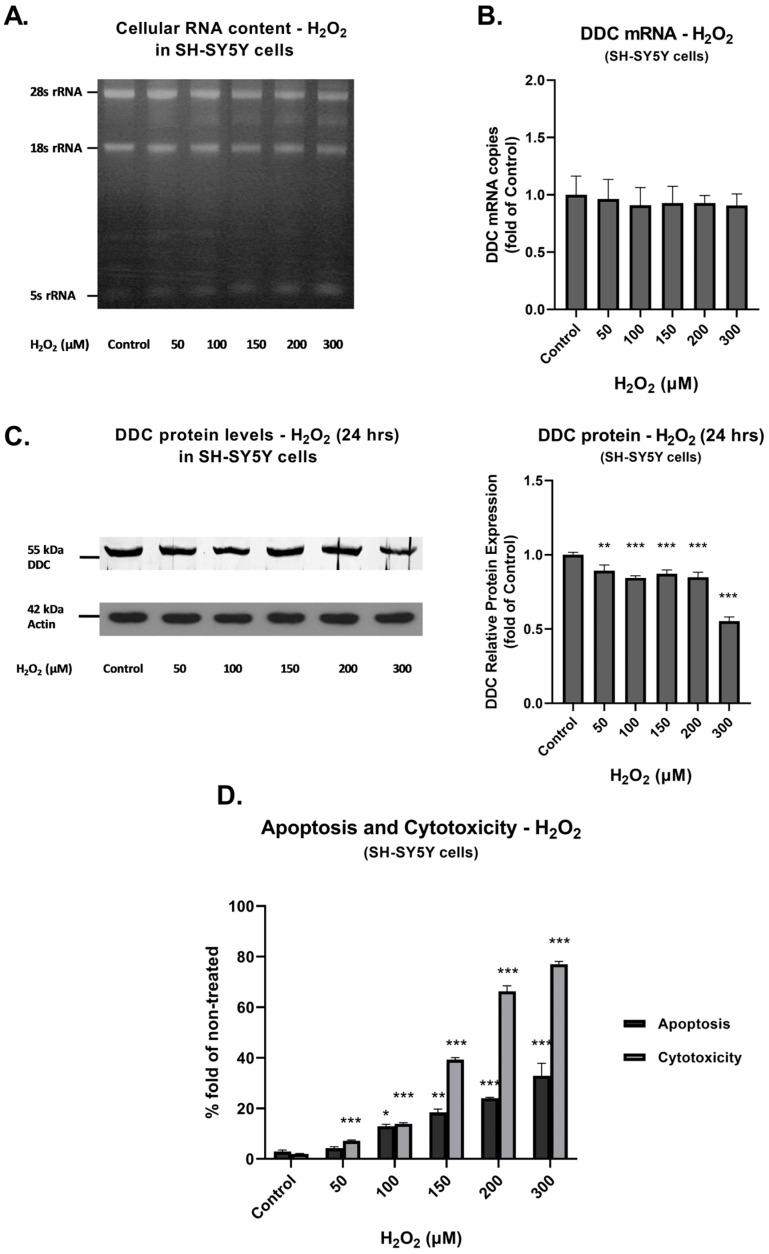
DDC expression, cytotoxicity, and apoptosis under conditions of prolonged oxidative stress in SH-SY5Y cells. (**A**) Agarose gel electrophoresis of extracted total RNA from cells exposed to H_2_O_2_ at the indicated concentrations for 24 h. (**B**) Relative DDC mRNA expression following exposure to H_2_O_2_ at the indicated concentrations for 24 h. Fold increase of DDC mRNA expression was measured using RT-qPCR in comparison to control (mock-treated) cells. Bars represent mean values from three separate experiments. Error bars represent standard deviation. (**C**) Western blot analysis performed using anti-DDC and anti-Actin antibodies in SH-SY5Y cells following exposure to H_2_O_2_ at the indicated concentrations for 24 h. Actin was used as a loading control. Graphs represent relative DDC protein expression against Actin. Bars depict mean values of three separate experiments. Error bars indicate standard deviations. ** *p* < 0.01 and *** *p* < 0.001 vs. control. (**D**) Effect of H_2_O_2_ on cytotoxicity (Grey bar) and apoptosis (Black bar): SH-SY5Y cells were exposed to H_2_O_2_ at the indicated concentrations for 24 h. Values presented are the percentages of cytotoxicity or apoptosis. Bars represent mean values from three separate experiments. Error bars represent standard deviation. * *p* < 0.05, ** *p* < 0.01, and *** *p* < 0.001 vs. control.

Figure 5 presents results obtained from the SH-SY5Y cell line. Figure 5A shows that degradation of the 28S rRNA was not as prominent as in the case of HEK-293 cells. Exposure to H_2_O_2_ did not result in significant differences in DDC mRNA expression (Figure 5B). Similarly, expression of full-length DDC protein remained stable in SH-SY5Y cells (Figure 5C). It is emphasized that, although DDC mRNA and protein expression levels remained constant, our data indicated statistically significant and high cytotoxicity and apoptosis levels. Peak cytotoxicity and apoptosis percentages were measured at 300 μM H_2_O_2_ corresponding to 77% (*p* < 0.001) and 32.9% (*p* < 0.001) (Figure 5D).

**Figure 6:**
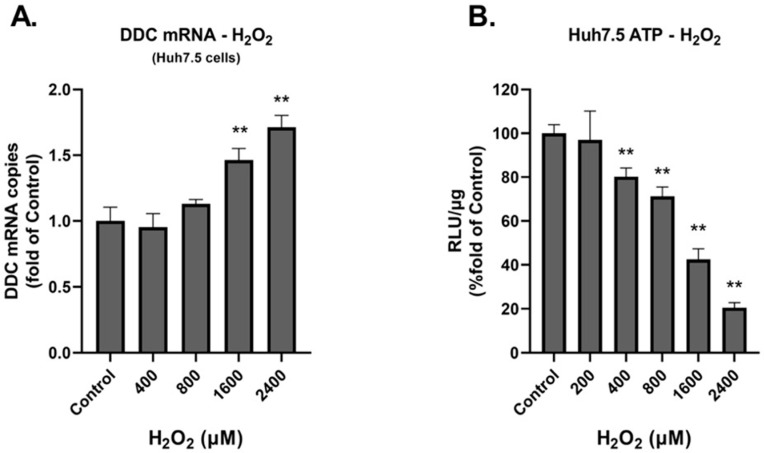
DDC expression and cytotoxicity under conditions of prolonged oxidative stress in Huh7.5 cells. (**A**) Relative DDC mRNA expression following exposure to H_2_O_2_ at the indicated concentrations for 24 h. Fold increase of DDC mRNA expression was measured using RT-qPCR in comparison to control (mock-treated) cells. DDC mRNA levels were normalized to YWHAZ mRNA. Data were obtained in triplicate from three independent experiments. ** *p* < 0.001 vs. control. (**B**) Huh7.5 cells were cultured for 24 h in the presence of H_2_O_2_ and 4 h prior to lysis H_2_O_2_ was replenished, or the cells were mock-treated (control). The intracellular ATP was measured (assessed as RLU/g of total protein) using a chemiluminescence-based assay. The ATP levels derived from the control cells were adjusted to 1. ** *p* < 0.001 vs. control.

Figure 6 exhibits results obtained from Huh7.5 cells. *DDC* mRNA levels were not significantly altered upon H_2_O_2_ exposure up to 800 μM (Figure 6A). However, in the higher concentrations of 1600 and 2400 μM, an elevation in *DDC* mRNA was evident. Subsequently, we tested the effect of H_2_O_2_ on cell viability in these cells, by assessing intracellular ATP levels (Figure 6B). We observed a dose-dependent reduction in the viability of the cells, in line with the cytotoxicity and apoptosis results attained from the HEK-293 and SH-SY5Y cells.

### 3.3. In DDC-Silenced Cells, the Effects of Prolonged Oxidative Stress Are more Pronounced

**Figure 7:**
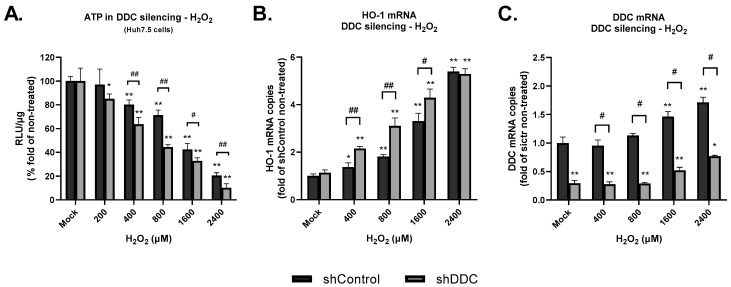
Effect of DDC silencing on H_2_O_2_-mediated cytotoxicity and oxidative stress. (**A**) Huh7.5 shControl/shDDC-expressing cells were cultured for 24 h in the presence of H_2_O_2_ and 4 h prior to lysis H_2_O_2_ was replenished, or the cells were mock-treated (mock). The intracellular ATP was measured (assessed as RLU/g of total protein) using a chemiluminescence-based assay. The ATP levels derived from the shControl mock-treated cells were adjusted to 100. * *p* < 0.05 and ** *p* < 0.001 vs. shControl mock and # *p* < 0.05 and ## *p* < 0.001 shDDC vs. shControl for each H_2_O_2_ concentration. (**B**,**C**) RT-qPCR was used to quantify HO-1 and DDC mRNA levels normalized to YWHAZ mRNA levels to shown transcription rates compared to mock-treated shControl-expressing cells (under normal growth conditions). Data were obtained in triplicate from three separate experiments. * *p* < 0.01 and ** *p* < 0.001 vs. shControl mock and # *p* < 0.01 and ## *p* < 0.001 shDDC vs. shControl for each H_2_O_2_ concentration.

Finally, in Huh7.5 cells, we examined the possibility that the negative impact of H_2_O_2_ on cell viability could be influenced by the absence of DDC. For this purpose, we performed DDC silencing in these cells (shDDC), as shown in Section 2, and applied the appropriate concentrations of H_2_O_2_ exogenously. DDC silencing had a negative effect on cell viability, as we observed reduced intracellular ATP levels at all applied H_2_O_2_ concentrations compared to shControl cells (Figure 7A). Correspondingly, the H_2_O_2_-mediated upregulation of the anti-oxidant marker HO-1 in the silenced cells was more pronounced, compared to the shControl cells (Figure 7B). We also determined DDC expression in both cell lines treated with concentrations of H_2_O_2_ similar to those used above. We observed that DDC expression was stable or upregulated (at the two higher concentrations of H_2_O_2_ applied), similarly between shControl and shDDC (Figure 7C). The above data suggest that expression of DDC is implicated in the cellular antioxidant mechanisms.

## 4. Discussion

Reactive oxygen species (ROS) are mainly produced in the mitochondrion as byproducts of cellular metabolism. Low levels of ROS are signal molecules in several biochemical processes [41]. At high concentrations, they cause damage to cell membranes and permanent DNA changes [42]. Excessive production of ROS or damage to cellular antioxidant mechanisms, both cause oxidative stress, which has been linked with several pathological conditions, including cancer, diabetes, and inflammatory and cardiovascular diseases [43]. In addition, oxidative stress has been found to be involved in the pathogenesis of numerous neurodegenerative illnesses, such as Parkinson’s (PD) and Alzheimer’s (AD) diseases, Huntington’s disease (HD), and amyotrophic lateral sclerosis (ALS) [6,43].

In this report, we show that DDC protein expression levels in human cells of neural and non-neural origin remain stable or increase, despite severe oxidative stress conditions. Furthermore, the present work provides evidence for a marked upregulation of *DDC* mRNA levels, as a result of oxidative stress in embryonic kidney HEK-293 and hepatoma Huh7.5 cells. DDC mRNA levels remain constant in neuroblastoma SH-SY5Y cells under oxidative stress conditions, despite total RNA degradation and considerable levels of cytotoxicity and apoptosis. Accordingly, DDC gene silencing resulted in increased cytotoxicity and *HO-1* gene expression upon H_2_O_2_ treatment, which could be attributed to oxidative stress conditions onset.

In PD, persistent loss of substantia nigra (SNpc) dopaminergic neurons leads to striatal dopamine (DA) depletion and Lewy body (LB) formation [44]. LBs are aggregates derived from the amassing of misfolded α-synuclein (α-Syn), a hallmark of Parkinson’s disease. However, the underlying mechanisms that are responsible for the fibrillation of α-Syn and the formation of LBs are not well understood [45]. It has been shown that intracellular DA is an inducer of the aggregation and stability of toxic α-Syn protofibrils. A-Syn has been found to act as a regulator of the quantity of DA in nerve terminals [46]. DA is considered a toxic neurotransmitter. It has been implicated in the pathogenesis of Parkinson’s disease [47]. The oxidative metabolism of DA is thought to cause the generation of significant amounts of ROS in dopaminergic neurons [48]. The autoxidation of DA in the presence of iron (Fe^2+^) and its oxidative deamination by monoamine oxidases MAO-A and MAO-B results to the production of ROS [49,50,51]. In addition, important factors in the reuptake of oxidation-prone cytoplasmic DA are the vesicular monoamine transporter (VMAT2) and the dopamine transporter (DAT) [52]. Both VMAT2 and DAT interact with α-Syn [53,54]. Although it is established that DA is instrumental in the mechanisms leading to Parkinson’s disease, not much is known about the involvement of the dopamine-synthesizing enzyme in neurodegeneration. Recently, DDC has been suggested as a biomarker of Parkinsonian disorders predicting the progression to clinical Lewy body disease [55]. Other published data showed that DDC modulates tau toxicity [24]. These investigators demonstrated that deletion in the *bas-1 DDC* gene of *C. elegans* ameliorated behavioral abnormalities in tau transgenic worms, reduced the accumulation of phosphorylated and detergent-insoluble tau, and decreased tau-mediated neuronal loss. These events led to a decrease in neurodegeneration.

With the aim of better understanding the biochemical regulatory mechanisms involving DDC, work by our team [19] has indicated the interaction between DDC and PI3K in human neuroblastoma, hepatocellular carcinoma, and embryonic kidney cells, as well as in mouse brain tissues. Phosphatidylinositol 3-kinase (PI3K) is involved in various biological processes. These include proliferation, metastasis, apoptosis, and angiogenesis [56,57]. Our results provided evidence for a possible regulatory role of DDC in pathways involving PI3K. Apoptosis is characterized by degradation of cytoplasmic mRNA and rRNA, as well as DNA damage and inhibited autophagy. We recently demonstrated that the key apoptosis marker Annexin V interacts with DDC, suggesting a role for DDC in apoptosis and programmed cell death molecular mechanisms [28]. These results led us to investigate aspects of the molecule’s role in oxidative stress, as they revealed DDC involvement in biochemical events that result in apoptosis and programmed cell death.

Here, we present evidence of DDC mRNA and protein expression under oxidative conditions in neural and nonneural human cell lines. Our data suggest that, at early stages of H_2_O_2_ exposure, production of ROS increased, whereas Western blot analysis showed that DDC protein expression remained stable or increased. Prolonged exposure to oxidizing factor resulted in degradation of total RNA, which was visible in both cases and was more pronounced in the nonneural cells. Moreover, cytotoxicity and apoptosis were induced in all cases. Interestingly, quantitative RT-PCR experiments showed different DDC mRNA expression levels when comparing the studied cell lines after exposure to the oxidant. In particular, HEK-293 and Huh7.5 cells showed H_2_O_2_-dependent upregulation of DDC mRNA expression, whereas no statistically significant differentiation in the expression of *DDC* mRNA in SH-SY5Y cells was observed, demonstrating impressive *DDC* mRNA stability despite very high cytotoxicity. In addition, Western blot analyses showed a high degree of stability of DDC protein expression. Taken together, these observations demonstrate the resistance of DDC to severe oxidative conditions and its possible involvement in oxidative stress mechanisms. Our observations suggest that there are two types of DDC expression patterns in response to oxidative stress. The resistance of DDC, which is independent of the increase in cytotoxicity and apoptosis, has also been described by other members of our research team [28,58]. Other proteins involved in neurodegeneration, like apolipoprotein E, amyloid precursor protein, and amyloid precursor-like protein 2, have also been shown to exhibit similar behavior, connecting their expression to the processes underlying cell death [59,60,61,62].

It is of interest to note that the silencing of the *DDC* gene caused an enhanced cytotoxicity upon H_2_O_2_-treatment, as shown by the lower cellular energy content compared to the normal culture conditions. Furthermore, suppression of DDC expression led to H_2_O_2_-mediated upregulation of the anti-oxidant marker HO-1, potentially implicating DDC silencing in enhanced oxidative stress conditions. Taken together, the above data indicate DDC has a role in the anti-oxidative mechanisms of the cell.

## 5. Conclusions

DDC has been referred to as a neglected and misunderstood enzyme. On the other hand, DDC has been shown to interact with PI3K [19] and be involved in the biochemical events leading to apoptosis [28]. The information obtained in this study showing the contribution of human DDC in oxidative stress highlights the critical importance of the enzyme under both physiological and pathological conditions. The significance of these expression patterns and the underlying regulatory mechanisms remain to be elucidated.

## Data Availability

All relevant data are within the manuscript.

## References

[1] Li Z., Wu J., Deleo C.J. (2006). RNA damage and surveillance under oxidative stress. IUBMB Life.

[2] Rahal A., Kumar A., Singh V., Yadav B., Tiwari R., Chakraborty S., Dhama K. (2014). Oxidative stress, prooxidants, and antioxidants: The interplay. BioMed Res. Int..

[3] Bienert G.P., Schjoerring J.K., Jahn T.P. (2006). Membrane transport of hydrogen peroxide. Biochim. Biophys. Acta.

[4] Stone J.R., Yang S. (2006). Hydrogen peroxide: A signaling messenger. Antioxid. Redox Signal..

[5] Dasuri K., Zhang L., Keller J.N. (2013). Oxidative stress, neurodegeneration, and the balance of protein degradation and protein synthesis. Free Radic. Biol. Med..

[6] Singh A., Kukreti R., Saso L., Kukreti S. (2019). Oxidative Stress: A Key Modulator in Neurodegenerative Diseases. Molecules.

[7] Bertoldi M. (2014). Mammalian Dopa decarboxylase: Structure, catalytic activity and inhibition. Arch. Biochem. Biophys..

[8] Lovenberg W., Weissbach H., Udenfriend S. (1962). Aromatic L-amino acid decarboxylase. J. Biol. Chem..

[9] Rahman M.K., Nagatsu T., Kato T. (1981). Determination of aromatic L-amino acid decarboxylase in serum of various animals by high-performance liquid chromatography with electrochemical detection. Life Sci..

[10] Lindström P., Sehlin J. (1983). Mechanisms underlying the effects of 5-hydroxytryptamine and 5-hydroxytryptophan in pancreatic islets. A proposed role for L-aromatic amino acid decarboxylase. Endocrinology.

[11] Craig S.P., Thai A.L., Weber M., Craig I.W. (1992). Localisation of the gene for human aromatic L-amino acid decarboxylase (DDC) to chromosome 7p13-->p11 by in situ hybridization. Cytogenet. Cell Genet..

[12] Ichinose H., Sumi-Ichinose C., Ohye T., Hagino Y., Fujita K., Nagatsu T. (1992). Tissue-specific alternative splicing of the first exon generates two types of mRNAs in human aromatic L-amino acid decarboxylase. Biochemistry.

[13] Albert V.R., Lee M.R., Bolden A.H., Wurzburger R.J., Aguanno A. (1992). Distinct promoters direct neuronal and nonneuronal expression of rat aromatic L-amino acid decarboxylase. Proc. Natl. Acad. Sci. USA.

[14] Sumi-Ichinose C., Hasegawa S., Ichinose H., Sawada H., Kobayashi K., Sakai M., Fujii T., Nomura H., Nomura T., Nagatsu I. (1995). Analysis of the alternative promoters that regulate tissue-specific expression of human aromatic L-amino acid decarboxylase. J. Neurochem..

[15] Jahng J.W., Wessel T.C., Houpt T.A., Son J.H., Joh T.H. (1996). Alternate promoters in the rat aromatic L-amino acid decarboxylase gene for neuronal and nonneuronal expression: An in situ hybridization study. J. Neurochem..

[16] Siaterli M.Z., Vassilacopoulou D., Fragoulis E.G. (2003). Cloning and expression of human placental L-Dopa decarboxylase. Neurochem. Res..

[17] Kokkinou I., Nikolouzou E., Hatzimanolis A., Fragoulis E.G., Vassilacopoulou D. (2009). Expression of enzymatically active L-DOPA decarboxylase in human peripheral leukocytes. Blood Cells Mol. Dis..

[18] Chalatsa I., Nikolouzou E., Fragoulis E.G., Vassilacopoulou D. (2011). L-Dopa decarboxylase expression profile in human cancer cells. Mol. Biol. Rep..

[19] Vassiliou A.G., Siaterli M.-Z., Frakolaki E., Gkogkosi P., Paspaltsis I., Sklaviadis T., Vassilacopoulou D., Vassilaki N. (2019). L-Dopa decarboxylase interaction with the major signaling regulator ΡΙ3Κ in tissues and cells of neural and peripheral origin. Biochimie.

[20] O’Malley K.L., Harmon S., Moffat M., Wong S., Uhland-Smith A. (1995). The human aromatic L-amino acid decarboxylase gene can be alternatively spliced to generate unique protein isoforms. J. Neurochem..

[21] Vassilacopoulou D., Sideris D.C., Vassiliou A.G., Fragoulis E.G. (2014). Identification and characterization of a novel form of the human L-dopaL-Dopa decarboxylase mRNA. Neurochem. Res..

[22] Adamopoulous P.G., Tsiakanikas P., Kontos C.K., Panagiotou A., Vassilacopoulou D., Scorilas A. (2019). Identification of novel alternative splice variants of the human L-DOPA decarboxylase (DDC) gene in human cancer cells, using high-throughput sequencing approaches. Gene.

[23] Paterson I.A., Juorio A.V., Boulton A.A. (1990). 2-Phenylethylamine: A modulator of catecholamine transmission in the mammalian central nervous system?. J. Neurochem..

[24] Kow R.L., Sikkema C., Wheeler J.M., Wilkinson C.W., Kraemer B.C. (2018). DOPA Decarboxylase Modulates Tau Toxicity. Biol. Psychiatry.

[25] Devos D., Lejeune S., Cormier-Dequaire F., Tahiri K., Charbonnier-Beaupel F., Rouaix N., Duhamel A., Sablonnière B., Bonnet A.-M., Bonnet C. (2014). Dopa-decarboxylase gene polymorphisms affect the motor response to L-dopa in Parkinson’s disease. Park. Relat. Disord..

[26] Li L., Lin H., Hua P., Yan L., Dong H., Li T., Liu W. (2020). Polymorphism of the Dopa-Decarboxylase Gene Modifies the Motor Response to Levodopa in Chinese Patients with Parkinson’s Disease. Front. Neurol..

[27] Frakolaki E., Kalliampakou K.I., Kaimou P., Moraiti M., Kolaitis N., Boleti H., Koskinas J., Vassilacopoulou D., Vassilaki N. (2019). Emerging Role of l-Dopa Decarboxylase in Flaviviridae Virus Infections. Cells.

[28] Chalatsa I., Arvanitis N., Arvanitis D., Tsakou A.C., Kalantzis E.D., Vassiliou A.G., Sideris D.C., Frakolaki E., Vassilaki N., Vassilacopoulou D. (2020). Human L-DopaL-Dopa decarboxylase interaction with annexin V and expression during apoptosis. Biochimie.

[29] Blight K.J., McKeating J.A., Rice C.M. (2002). Highly Permissive Cell Lines for Subgenomic and Genomic Hepatitis C Virus RNA Replication. J. Virol..

[30] Mpekoulis G., Tsopela V., Chalari A., Kalliampakou K.I., Panos G., Frakolaki E., Milona R.S., Sideris D.C., Vassilacopoulou D., Vassilaki N. (2022). Dengue Virus Replication Is Associated with Catecholamine Biosynthesis and Metabolism in Hepatocytes. Viruses.

[31] Kalajzic I., Stover M., Liu P., Kalajzic Z., Rowe D., Lichtler A. (2001). Use of VSV-G pseudotyped retroviral vectors to target murine osteoprogenitor cells. Virology.

[32] Dull T., Zufferey R., Kelly M., Mandel R.J., Nguyen M., Trono D., Naldini L. (1998). A third-generation lentivirus vector with a conditional packaging system. J. Virol..

[33] Eruslanov E., Kusmartsev S. (2010). Identification of ROS using oxidized DCFDA and flow-cytometry. Methods Mol. Biol..

[34] Louis K.S., Siegel A.C. (2011). Cell viability analysis using trypan blue: Manual and automated methods. Methods Mol. Biol..

[35] Gibb R.K., Gercel-Taylor C. (2001). Use of diphenylamine in the detection of apoptosis. Methods Mol. Med..

[36] Livak K.J., Schmittgen T.D. (2001). Analysis of relative gene expression data using real-time quantitative PCR and the 2^−ΔΔC_T_^ Method. Methods.

[37] Bradford M.M. (1976). A rapid and sensitive method for the quantitation of microgram quantities of protein utilizing the principle of protein-dye binding. Anal. Biochem..

[38] Laemmli U.K. (1970). Cleavage of structural proteins during the assembly of the head of bacteriophage T4. Nature.

[39] Towbin H., Staehelin T., Gordon J. (1979). Electrophoretic transfer of proteins from polyacrylamide gels to nitrocellulose sheets: Procedure and some applications. Proc. Natl. Acad. Sci. USA.

[40] Batteiger B., Newhall W.J., Jones R.B. (1982). The use of Tween 20 as a blocking agent in the immunological detection of proteins transferred to nitrocellulose membranes. J. Immunol. Methods.

[41] Moreno-Loshuertos R., Acín-Pérez R., Fernández-Silva P. (2006). Differences in reactive oxygen species production explain the phenotypes associated with common mouse mitochondrial DNA variants. Nat. Genet..

[42] Paravicini T.M., Touyz R.M. (2020). Redox signaling in hypertension. Cardiovasc. Res..

[43] Yang S., Lian G. (2020). ROS and diseases: Role in metabolism and energy supply. Mol. Cell Biochem..

[44] Fahn S., Sulzer D. (2014). Neurodegeneration and neuroprotection in Parkinson disease. NeuroRx.

[45] Mahul-Mellier A.-L., Burtscher J., Maharjan N., Weerens L., Croisier M., Kuttler F., Leleu M., Knott G.W., Lashuel H.A. (2020). The process of Lewy body formation, rather than simply α-synuclein fibrillization, is one of the major drivers of neurodegeneration. Proc. Natl. Acad. Sci. USA.

[46] Conway K.A., Rochet J.C., Bieganski R.M., Lansbury P.T. (2001). Kinetic stabilization of the alpha-synuclein protofibril by a dopamine-alpha-synuclein adduct. Science.

[47] Caudle W.M., Colebrooke R.E., Emson P.C., Miller G.W. (2008). Altered vesicular dopamine storage in Parkinson’s disease: A premature demise. Trends Neurosci..

[48] Westlund K.N., Denney R.M., Rose R.M., Abell C.W. (1988). Localization of distinct monoamine oxidase A and monoamine oxidase B cell populations in human brainstem. Neuroscience.

[49] Muñoz P., Huenchuguala S., Paris I., Segura-Aguilar J. (2012). Dopamine oxidation and autophagy. Park. Dis..

[50] Hare D.J., Double K.L. (2016). Iron and dopamine: A toxic couple. Brain.

[51] Trist B.G., Hare D.J., Double K.L. (2019). Oxidative stress in the aging substantia nigra and the etiology of Parkinson’s disease. Aging Cell.

[52] Dias V., Junn E., Mouradian M.M. (2013). The role of oxidative stress in Parkinson’s disease. J. Park. Dis..

[53] Dean E.D., Li Y., Torres G.E., Miller G.W. (2008). Identification of a novel interaction between α-synuclein and VMAT2. FASEB J..

[54] Butler B., Goodwin S., Saha K., Becker J.P., Sambo D., Davari P., Goodwin J.S., Khoshbouei H. (2015). Dopamine transporter activity is modulated by α-synuclein. J. Biol. Chem..

[55] Pereira J.B., Kumar A., Hall S., Palmqvist S., Stomrud E., Bali D., Parchi P., Mattsson-Carlgren N., Janelidze S., Hansson O. (2023). DOPA decarboxylase is an emerging biomarker for Parkinsonian disorders including preclinical Lewy body disease. Nat. Aging.

[56] Yuan Y.U. (2014). Role of PI3K/Akt/mTOR signaling pathway in hepatocellular carcinoma. Lin. Chuang Gan Dan. Bing. ZaZhi.

[57] Glaviano A., Foo A.S.C., Lam H.Y., Yap K.C.H., Jacot W., Jones R.H., Eng H., Nair M.G., Makvandi P., Geoerger B. (2023). PI3K/AKT/mTOR signaling transduction pathway and targeted therapies in cancer. Mol. Cancer.

[58] Kalantzis E.D., Scorilas A., Vassilacopoulou D. (2018). Evidence for L-Dopa Decarboxylase Involvement in Cancer Cell Cytotoxicity Induced by Docetaxel and Mitoxantrone. Curr. Pharm. Biotechnol..

[59] Araki W., Wurtman R.J. (1998). Increased expression of amyloid precursor protein and amyloid precursor-like protein 2 during trophic factor withdrawal-induced death of neuronal PC12 cells. Brain Res. Mol. Brain Res..

[60] Quinn C.M., Kågedal K., Terman A., Stroikin U., Brunk U.T., Jessup W., Garner B. (2001). Induction of fibroblast apolipoprotein E expression during apoptosis, starvation-induced growth arrest and mitosis. Neuropharmacology.

[61] Strope T.A., Wilkins H.M. (2023). Amyloid precursor protein and mitochondria. Curr. Opin. Neurobiol..

[62] Loch R.A., Wang H., Perálvarez-Marín A., Berger P., Nielsen H., Chroni A., Luo J. (2023). Cross interactions between Apolipoprotein E and amyloid proteins in neurodegenerative diseases. Comput. Struct. Biotechnol. J..

